# Goitre

**Published:** 1917-09

**Authors:** Leigh F. Watson

**Affiliations:** Chicago


### Journal-Record of Medicine

Successor to Atlanta Medical and Surgical Journal, Established 1855
and Southern Medical Record, Established 1870

**OWNED BY THE ATLANTA MEDICAL JOURNAL COMPANY**

PUBLISHED MONTHLY

*Official Organ Fulton County Medical Society, State Examing Board, Atlanta, Birmingham and Atlantic Railroad Surgeon's Association, Chattahoochee Valley Medical and Surgical Association, Etc.*

A. W. STIRLING, M. D., C. M., D. P. H.; J. S. HURT, B. Ph., M.D.
GEO. M. NILES, M. D., W. J. LOVE, M. D., (Ala.); Associate Editors

E. W. ALLEN, Business Manager

**COLLABORATORS**

W. F. WESTMORELAND, M. D., General Surgery
F. W. McRAE, M. D., Abdominal Surgery
ALLEN H. BUNCE, Medical Societies
E. B. BLOCK, M. D., Diseases of the Nervous System
MICHAEL HOKE, M. D., Orthopedic Surgery
CYRUS W. STRICKLER, M. D., Legal Medicine and Medical Legislation
E. C. DAVIS, A. B., M. D., Obstetrics
E. G. JONES, A. B., M. D., Gynecology
ALLEN H. BUNCE, M. D., Pathology and Bacteriology
R. T. DORSEY, Jr., B. S., M. D., Medicine
L. M. GAINES, A. B., M. D., Internal Medicine
GEO. C. MIZELL, M. D., Diseases of the Stomach and Intestines
L. B. CLARKE, M. D., Pediatrics
EDGAR PAULLIN, M. D., Opsonic Medicine
THEODORE TOEPEL, M. D., Mechano Therapy
E. G. BALLENGER, M. D., Diseases of the Genito-Urinary Organs

VOL. LXIV. ATLANTA, GA., SEPT., 1917. NO. 6

### GOITRE.

### An Analysis of 125 Cases with a Note on the Treatment.

By Leigh F. Watson, M.D., Chicago

An Abstract:—

The author reviews the records of 125 goitre patients considering the cause, age at onset, and effect of previous opera tion in certain cases. He illustrates by tables the degree of enlargement, and reports the results following quinin and urea injection.

In 43 per cent. no exiciting cause could be elicited; in the remaining 57 per cent. the onset could be ascribed to a definite exciting cause. Of the 125 cases, 15 per cent. was caused by worry; parturition was responsible for 11 per cent., and in 9 per cent. the condition was due to puberty. Twenty per cent. gave a family history of goiter and 11 per cent of nervousness; 19 per cent. had had tonsillitis. Forty-five per cent. of the exopthalmic patients first noted the goiter eight years before examination at the average age of 34 years, and the symptoms developmen at the age of 40. Fifty per cent. gave a history of acute onset, two years before coming under observation at the average age of 29 years. Sixty per cent. of the nonexopthalmic patients observed that they developed more marked symptoms of intoxication as the goiter became more chronic.

Before coming under treatment, five exopthalmic patients had had ligation of the superior thyroid arteries with temporary relief; four had had partial thyroidectomies without permanent benefit; three had had pelvic operations without lessening the hyperthyroidism; the condition of one was aggravated by a panhysterectomy; and one had had a tonsillectomy six months before without influencing the severity of the exopthalmic symptoms. Enlargement usually begins in the right lobe, sometimes in the isthmus and least frequently in the left lobe. In 95 per cent. of the exopthalmic patients of this group both lobes and isthmus were involved before the goiter became exopthalmic. A majority of the patients noticed increasing symptoms of intoxication as the goiter became more chronic, gradually involving both lobes and isthmus. Eighteen per cent. of the mildly toxic patients became exopthalmic after an average period of five years. This study indicates that both nontoxic and toxic goiter occur later in life in nongoitrous localities than in sections where the disease is more prevalent.

The following tables show the results after quinin and urea injection:

| Effect of Injection on Symptons. | Relieved | Improved | Not Imp. |
|---|---|---|---|
| Exopthalmic | 85 (aver. 4 mos.) | 15 | 0 |
| Nonexapthalmic | 84 (aver. 4 mos.) | 10 | 0 |

| Effect of Injections on Goiter | Cured | Reduced | Not Red. |
|---|---|---|---|
| Exopthalmic | 80 (aver. 5 mos.) | 15 | 5 |
| Nonexopthalmic | 75 (aver. 4 mos.) | 12 | 13 |

Two patients suffering with sever toxic goiter with exopthalmos of several years duration received only slight benefit; later a lobectomy was done without addition relief. Four exopthalmic patients were pregnant two to four months. Relief from hyperthyroidism followed the injection and they went to term without recurrenct and had normal deliveries. The number of patients cured is highest in the group of those who came for treatment early in the disease; the benefit received by those who came later was in proportion to the degree of damage done the circulatory and nervous systems. A goiter that has once disappeared has never recurred. A majority of the patients in this group have been under observation from two to four years. The quinin and urea injection has limitations the same as any other treatment for goiter and can be employed only in selected cases. The treatment of the exopthalmic type in young adults is very difficult, and should be attempted only under the most favorable circumstances. If the best results are to be secured, hyperthyroidal patients must have at least a year of mental and physical rest after treatment.

